# Genetic Dissection of Drought and Heat Tolerance in Chickpea through Genome-Wide and Candidate Gene-Based Association Mapping Approaches

**DOI:** 10.1371/journal.pone.0096758

**Published:** 2014-05-06

**Authors:** Mahendar Thudi, Hari D. Upadhyaya, Abhishek Rathore, Pooran Mal Gaur, Lakshmanan Krishnamurthy, Manish Roorkiwal, Spurthi N. Nayak, Sushil Kumar Chaturvedi, Partha Sarathi Basu, N. V. P. R. Gangarao, Asnake Fikre, Paul Kimurto, Prakash C. Sharma, M. S. Sheshashayee, Satoshi Tobita, Junichi Kashiwagi, Osamu Ito, Andrzej Killian, Rajeev Kumar Varshney

**Affiliations:** 1 International Crops Research Institute for the Semi-Arid Tropics (ICRISAT), Hyderabad, Andhra Pradesh, India; 2 Guru Gobind Singh Indraprastha University, Delhi, India; 3 Indian Institute of Pulses Research (IIPR), Kanpur, Uttar Pradesh, India; 4 International Crops Research Institute for the Semi-Arid Tropics (ICRISAT), Nairobi, Kenya; 5 Ethiopian Institute of Agricultural Research (EIAR), Debre Zeit, Ethiopia; 6 Egerton University (EU), Egerton, Kenya; 7 University of Agricultural Sciences- Bangalore, Bangalore, Karnataka, India; 8 Japan International Research Center for Agricultural Sciences (JIRCAS), Tsukuba, Japan; 9 Hokkaido University, Sapporo, Japan; 10 United Nations University, Yokohama, Japan; 11 DArT Pty. Ltd., Yarralumla, Australia; Institute for Sustainable Agriculture (IAS-CSIC), Spain

## Abstract

To understand the genetic basis of tolerance to drought and heat stresses in chickpea, a comprehensive association mapping approach has been undertaken. Phenotypic data were generated on the reference set (300 accessions, including 211 mini-core collection accessions) for drought tolerance related root traits, heat tolerance, yield and yield component traits from 1–7 seasons and 1–3 locations in India (Patancheru, Kanpur, Bangalore) and three locations in Africa (Nairobi, Egerton in Kenya and Debre Zeit in Ethiopia). Diversity Array Technology (DArT) markers equally distributed across chickpea genome were used to determine population structure and three sub-populations were identified using admixture model in STRUCTURE. The pairwise linkage disequilibrium (LD) estimated using the squared-allele frequency correlations (r^2^; when r^2^<0.20) was found to decay rapidly with the genetic distance of 5 cM. For establishing marker-trait associations (MTAs), both genome-wide and candidate gene-sequencing based association mapping approaches were conducted using 1,872 markers (1,072 DArTs, 651 single nucleotide polymorphisms [SNPs], 113 gene-based SNPs and 36 simple sequence repeats [SSRs]) and phenotyping data mentioned above employing mixed linear model (MLM) analysis with optimum compression with P3D method and kinship matrix. As a result, 312 significant MTAs were identified and a maximum number of MTAs (70) was identified for 100-seed weight. A total of 18 SNPs from 5 genes (*ERECTA*, 11 SNPs; *ASR*, 4 SNPs; *DREB*, 1 SNP; *CAP2* promoter, 1 SNP and *AMDH*, 1SNP) were significantly associated with different traits. This study provides significant MTAs for drought and heat tolerance in chickpea that can be used, after validation, in molecular breeding for developing superior varieties with enhanced drought and heat tolerance.

## Introduction

Chickpea (*Cicer arietinum* L) is the second most important grain legume cultivated mostly on residual soil moisture in the arid and semi-arid regions of the world. It is a diploid member of family Leguminosae with basic chromosome number eight and genome size 738 Mb [1]. Globally it is cultivated on over 13.2 Mha with an annual production of 11.6 million metric tons [2]. Chickpea is a rich source of proteins, essential amino acids and vitamins such as riboflavin, niacin, thiamin, folate and the vitamin A precursor β-carotene [3]. Based on seed size and color chickpea is grouped into two market classes namely *desi* and *kabuli*. Globally about 80% of total production is contributed by *desi* cultivars. Further, among chickpea growing countries India alone contributes to 70% of the world's total production [2]. Other major chickpea producing countries include Pakistan, Turkey, Australia, Myanmar, Ethiopia, Iran, Mexico, Canada and the USA. Although the chickpea production potential is high, it is not fully realized owing to several abiotic and biotic stresses. Among abiotic stresses that affect the chickpea production, drought and heat are considered as major constraints. Annually, 40–50% reduction in yield has been reported worldwide as a result of terminal drought in chickpea [4]. Further, the damage due to drought is compounded by heat stress in the warmer Mediterranean regions and regions like South Asia where temperature increases towards flowering [5] and it is difficult to differentiate between the damage caused by the individual stresses. Nevertheless as a result of drought stress, the growing season may be shortened affecting yield components, i.e., total biomass, pod number, seed number, seed weight and quality and yield plant^−1^ [6]. Flowering and seed set are the most critical growth stages affected by drought in chickpea. Drastic reductions in chickpea seed yields were observed when plants were exposed to high (35°C) temperatures at flowering and pod development stages [7]. Heat stress also adversely affects pollen viability, fertilization and seed development leading to a reduced harvest index. The identification of genomic regions associated with the drought and heat tolerance would enable breeders to develop improved cultivars with increased drought and heat tolerance using molecular breeding.

Large scale genomic resources are essential for understanding the genetics of complex abiotic stresses like drought, heat tolerance etc. In the case of chickpea, during last five years >3,000 microsatellites or simple sequence repeats (SSRs) [8]–[10], Diversity Array Technology (DArT) arrays [10] and single nucleotide polymorphism (SNP) [11] markers were developed. Further these marker resources were used for linkage map construction [9], [10] as well as trait mapping. Majority of trait mapping studies were focused on biotic stresses [12]. Recently sequencing of *desi* and *kabuli* chickpea genomes has been completed [1], [13] and a genome-wide physical map (http://probes.pw.usda.gov:8080/chickpea/) was developed [14]. Despite the availability of large scale genomic resources, most of the studies to understand the genetics of complex traits were limited to quantitative trait loci (QTL) mapping studies. Moreover, the family based QTL studies were mostly limited to biotic stresses like *Fusarium* wilt, *Ascochyta* blight and *Botrytis* gray mold [12]. Very few studies were conducted to understand the genetics of drought tolerance [15] and salt tolerance [16] in chickpea.

Despite the continuous efforts to enhance the productivity of chickpea, climate changes during past two decades had tremendous influence on the production and productivity [17]. Global warming, coupled with increased temperatures in arid and semi-arid regions has necessitated development of crop varieties that can sustain and yield high in harsh climatic conditions by virtue of being resilient to warmer temperatures. Further, the quantitative inheritance of drought and heat; their interaction with environment have been posing challenges to our understanding of the genetic basis of these traits. Although conventional breeding has substantial impact in marginal chickpea growing environments [18], future genetic gains will require a more systematic use of physiological and genetic approaches, facilitated by the rapid increase in genome knowledge and understanding. Thus the knowledge generated through advances in genomics during past two decades, have enormous potential in enhancing the tolerance to these stresses.

During last two decades, molecular markers have provided greater insights into complex traits in several crop species and the research endeavors in crop improvement shifted from quantitative to molecular genetics with emphasis on QTL identification and adoption of marker-assisted selection (MAS). However, only modest results have been witnessed due to several factors including absence of tight linkage between makers and QTLs, non-availability of mapping populations, and substantial time needed to develop such populations. Further, the QTL mapping approaches cannot make use of the huge variation present across the germplasm available in the genebanks. In addition, the resolution achieved through linkage mapping based on bi-parental mapping population is low compared to population linkage disequilibrium (LD) based association mapping. The genome-wide association study (GWAS) makes it possible to simultaneously screen a very large number of accessions for genetic variation underlying diverse complex traits. In fact, the association studies in other crop species especially in cereals [19]–[21] have revealed that the linkage based QTL analyses can be complemented by LD based association studies. Association mapping studies in legumes are limited to soybean [22], *Medicago* [23] and common bean [24]. Most of these association studies either have deployed GWAS or candidate gene-sequencing approach. In some recent studies, however, the combined approach of GWAS and candidate-gene sequencing has been shown as more powerful approach than the individual approach [25]. However, to date there is no report on association studies in the case of chickpea. Recently a diverse set of 300 accessions, called as ‘reference set’ [26] that included ‘mini core collection’ [27] has been used to analyze sequence diversity for 10 drought responsive genes [28].

In the present study, both genome-wide and candidate gene-sequencing based association mapping approaches were employed to understand genetics of the two most important complex abiotic stresses “drought” and “heat” and establish significant marker-trait associations (MTAs). For this the chickpea reference set/mini-core collection was genotyped for 1,813 marker loci (SSRs, DArTs and SNPs) and phenotyped at three locations in Africa (Nairobi and Egerton in Kenya; and Debre Zeit in Ethiopia) and three locations in India (Patancheru, Kanpur and Bangalore). Besides significant MTAs, the present study also provides an in-depth understanding of the genetic diversity, population structure in the reference set and linkage disequilibrium (LD) in genome of chickpea.

## Results and Discussion

The chickpea reference set, comprising of 300 diverse accessions of *Cicer* spp. including 293 accessions from *C. arietinum*, 4 accessions from *C. reticulatum* and 3 accessions from *C. echinospermum* (Table S1), from Asia (198), Africa (21), Europe (3), Mediterranean (56), Americas (10), CIS (6), and 6 accessions with unknown geographical origin, was chosen for association studies. This set was evaluated for 34 traits (root, morphological, phenological, transpiration efficiency related traits, yield and yield related traits) under drought and heat stress environments with 2–3 replications and 1–7 years (Table S2). Large phenotypic variation was observed for all traits phenotyped on the reference set or mini-core collection (Table S3).

### Genome-wide marker profiling

We report the first comprehensive characterization of the chickpea reference set using 16,046 markers (35 SSRs, 15,360 DArT features and 651 SNPs) in the present study. A total of 917 alleles were detected by 35 SSR markers with an average of 26.2 alleles/marker locus and the polymorphism information content (PIC) values ranged from 0.48–0.96 (Table S4). Further, major allele frequency and the overall mean heterozygosity was 0.21 and 0.04 respectively. The DArT markers developed by Thudi et al. [10] were used for genotyping the reference set, as a result 1,156 DArT loci were found polymorphic with PIC values in the range of 0.01–0.38 (Table S5). Among 651 SNPs genotyped using KASPar assays, 381 SNPs were polymorphic and the PIC values ranged from 0.007–0.375 (Table S6).

### Allele mining in candidate drought responsive genes

A total of 10 candidate drought responsive genes (Table S7) were amplified on the reference set/mini-core collection. Of these, 5 genes (Abscisic acid stress and ripening, *ASR*; *CAP2* gene; *ERECTA*; sucrose synthase, *SUSY*; Sucrose phosphate synthase, *SPS*) were amplified on the reference set. The number of genotypes for which good quality sequences were obtained varied from 79 (*ERECTA* fragment obtained from 7f–5r primer pairs) to 236 genotypes (*SPS* gene) out of 300 genotypes attempted [28]. The longest sequence obtained was for *SUSY* gene which was about 1,600 bp and the shortest sequence was obtained for *SPS* gene with 312 bp. Varying number of SNPs were identified in each gene using *DIVEST* tool. SNPs were inspected manually for possible sequencing errors and SNPs having clear peaks were considered as true SNPs. For instance, highest number of SNPs (34) was obtained for *ASR* gene while no SNPs were found in the case of *CAP2* gene. In total, 33 SNPs were identified in case of *ERECTA* gene, of which 13 SNPs (9 transitions and 4 transversions) were in *ERECTA* (7f–5r) gene fragment and 20 SNPs (10 transitions and 10 transversions) in case of *ERECTA* (8f–8r) gene fragment. Only 1 indel was observed in case of *ERECTA* (7f–5r) gene fragment. One indel and 3 SNPs were observed in case of *SPS* gene sequence (Table S7). In addition to above mentioned 5 drought responsive genes, 5 abiotic stress responsive genes (AKIN- SNF1 related protein kinase, *AKIN*; Aminoaldehyde dehydrogenase gene, *AMADH*; Dehydrin, *DHN*; Dehydration responsive element binding protein, *DREB* and Myb transcription factor, *Myb*) were used for allele mining across mini-core collection (Table S7). Out of 211 genotypes screened, number of genotypes yielding high quality sequence varied from 191 (*DREB*) to 209 (*AMADH*). Highest number of SNPs (14) was obtained for *DREB* gene (8 transitions and 6 transversions). Apart from SNPs, 23 indels were also detected. *AKIN* gene was found to be most conserved with just 2 SNPs (transitions) and 2 indels. A total of 13 SNPs were identified (6 transitions and 7 transversions) in case of *AMADH* with 3 indels among 209 high quality sequences, while in case of *DHN* gene 7 SNPs (5 transitions and 2 transversions) were identified among 198 sequence analyzed. For *Myb* gene only 6 SNPs (1 transition and 5 transversions) were reported with 2 indels across *Myb* sequences. Average PIC value of SNPs ranged from 0 (*CAP2* gene) to 0.43 (*CAP2* promoter) (Table S7).

### Population structure and genetic relationships

**Figure 1:**
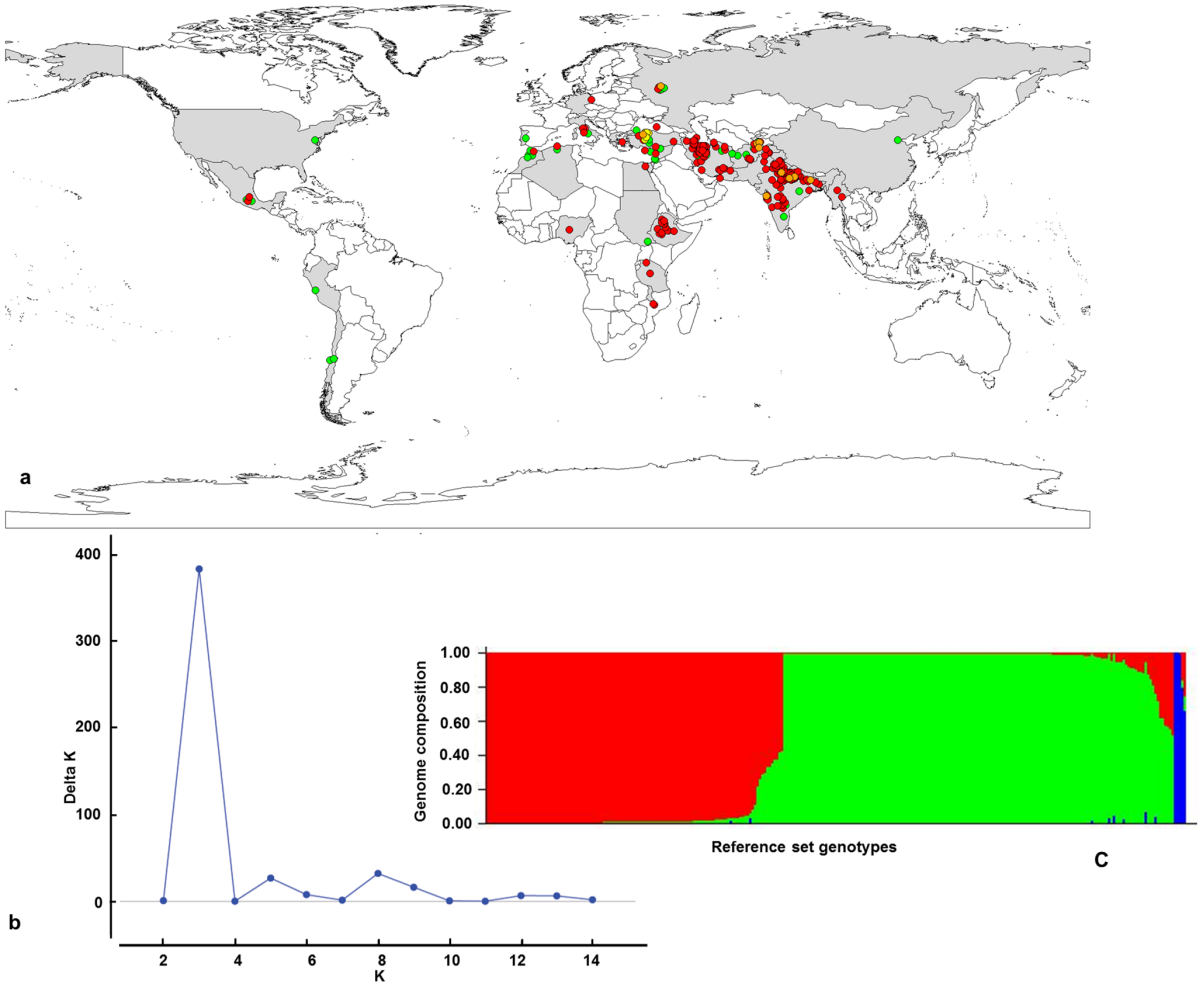
Geographic origin and population structure of chickpea reference set. a) the distribution of chickpea reference set, *desi* in red, *kabuli* in green, pea-shaped in orange and wild in yellow color dots b) ΔK is function of k from the structure run, the plateau at k = 3 indicates number of sub-populations in the reference set; c) Clustering of chickpea set genotypes into three groups (Group I, Group II and Group III).

In order to assess the population structure and number of sub-populations, 85 evenly distributed DArT loci on chickpea genome [10] were used on the reference set employing STRUCTURE 2.3.4 [29]. Based on the maximum likelihood and delta K (ΔK) values, three sub-populations (Group I, Group II and Group III) were determined in the reference set (Fig 1). However, different K values are possible (Fig S1); nevertheless, these do not qualitatively affect the results. As the K value is increasing the allelic admixture among the sub-populations is more clearly demarcated. Using a membership probability threshold of 0.60, 109 accessions were assigned to sub-population 1; 154 accessions to sub-population 2; 26 accessions to sub-population 3 and 11 accessions were retained in the admixed group (Table S1).

To understand the genetic diversity in the reference set neighbor joining (NJ) trees were constructed using allelic data from 35 SSRs, 1,156 DArT loci and 651 SNP markers independently as well as all markers combined together, which revealed three major clusters (Fig. S1). However, the grouping of the accessions in each cluster for different marker systems was not similar. This can be attributed to the nature of markers used and the genomic regions sampled by these marker systems. Nevertheless, the SNP markers could more clearly demarcate the accessions into smaller sub-groups compared to SSRs and DArT loci (Fig S1). This indicates SNP markers have more potential application for plant genome analysis compared to other markers. However, none of the three marker systems could group the association panel into distinct groups based on either the market class or the biological status of the germplasm set analyzed. This indicates that genetic variation in the reference set is very high and it is an ideal germplasm panel for association studies. The wild genotypes were grouped in third cluster and these are distributed away from the cultivated genotypes in the cluster indicating that the wild species genotypes are more distinct (Fig S1).

### Linkage disequilibrium (LD) decay

**Figure 2:**
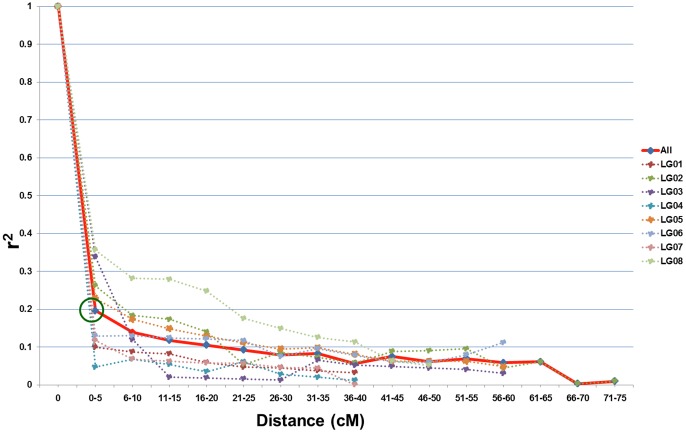
Linage disequilibrium (LD) decay across all linkage groups. The overall LD decay across the genome is at 5

A consensus genetic map was constructed using the two inter-specific genetic maps developed by [10] and [11] based on mapping population ICC 4958× PI 489777. A total of 1,358 markers (706 DArT loci, 622 SNPs and 30 SSRs) mapped on to the consensus map were used for estimating the LD decay. Estimation of LD decay is essential to determine the number of markers required for association mapping of complex traits like drought and heat tolerance in chickpea. In general, the genetic distance at which r^2^ decays to 0.1–0.2 is considered to be the extent of LD in a species [30]. In the present study, the pairwise LD estimated using the squared-allele frequency correlations (r^2^; when r^2^<0.20) was found to decay rapidly with the genetic distance of 5 cM (Fig 2), when r^2^<0.1 LD decay was found to decay at a genetic distance of 20 cM. The results suggest that, LD decay over short distances will facilitate fine mapping of QTL, while LD decay over longer distances will facilitate initial association of trait data with haplotypes in chromosome regions. Further, researchers can use the LD map as a reference to find target QTL and genes for positional cloning [31].

### Genome-wide and candidate gene-sequencing based association

In order to reduce the number of false positive associations, both population structure and relative kinship information were employed. Mixed linear model (MLM) with optimum compression and P3D in TASSEL 2.01 software was employed for establishing MTAs. Further, to eliminate the false positives Bonferroni correction was used and as a result, a total of 312 highly significant MTAs were identified for 25 agronomically important traits (Table 1). Phenotypic variance explained (PVE) for these MTAs ranged from low (4.14%) to very high (96.55%) and thus MTAs detected with high PVE for desired traits can be improved through molecular breeding.

**Table 1:** Significant marker-trait associations (MTAs) identified for different traits

Trait	Number of marker trait associations	P- value range	Phenotypic variation (%)
**Root traits**			
Root dry weight (RDW, g plant^−1^)	2	1.16×10^−6^–3.67×10^−6^	8.25–10.344
Root length density (RLD, cm cm^−3^)	2	2.89×10^−10^–9.19×10^−8^	9.96–11.17
Root surface area (RSA, cm^2^ plant^−1^)	2	5.73×10^−7^–7.41×10^−7^	11.95–21.71
Root volume (RV, cm^3^ plant^−1^)	2	2.80×10^−6^–2.87×10^−6^	10.60–19.74
Rooting depth (RDp, cm)	7	1.56×10^−11^–1.15×10^−8^	13.12–22.41
**Morphological traits**			
Plant height (PHT, cm)	35	2.48×10^−14^–4.58×10^−6^	12.76–38.01
Shoot dry weight (SDW, g)	3	5.45×10^−11^–2.09×10^−6^	7.77–19.61
Apical secondary branches	5	3.46×10^−8^–1.97×10^−7^	12.36–17.38
Basal primary branches	4	1.44×10^−10^–1.34×10^−6^	11.53–19.44
**Phenological traits**			
Days to maturity (DM)	5	9.84×10^−17^–3.81×10^−6^	4.14–79.31
Flowering days (FD)	2	1.71×10^−34^–1.61×10^−6^	11.49–96.55
**Yield related traits**			
100-seed weight (100SDW, g)	70	9.53×10^−14^–9.77×10^−6^	8.73–36.95
Biomass (BM)	11	8.43×10^−10^–1.22×10^−8^	16.34–18.99
Harvest index (HI, %)	16	5.33×10^−17^–8.24×10^−7^	4.23–15.53
Yield (YLD)	32	6.09×10^−16^–5.09×10^−6^	11.43–29.03
Pod m^−2^ (POD)	10	3.27×10^−8^–1.86×10^−6^	9.18–22.05
Pods plant^−1^ (PPP)	6	8.12×10^−19^–3.26×10^−6^	8.27–50.44
Seed m^−2^ (SPM)	34	4.02×10^−12^–4.73×10^−6^	8.06–55.42
Seed pod^−1^ (SPP)	13	2.82×10^−12^–4.85×10^−6^	7.72–17.75
Per day shoot (PDS)	1	1.22×10^−6^	10.09
Production (PROD)	3	8.12×10^−19^–3.26×10^−6^	18.00–50.44
Heat tolerance index (HTI)	9	1.77×10^−12^–3.42×10^−6^	11.17–30.59
Rate of partitioning coefficient	7	5.19×10^−8^–4.32×10^−6^	5.03–14.99
Total dry matter weight (TDM, g/m^2^)	9	2.01×10^−8^–4.56×10^−6^	8.84–12.88
**Transpiration efficiency**			
Delta Carbon ratio (δ^13^C)	22	2.59×10^−16^–4.68×10^−6^	7.81–34.77
Total MTAs	312		

#### Drought tolerance root traits

Drought is the major limiting factor to crop production and especially chickpea experiences various kinds of drought stresses depending on the timing and intensity of the water stress relative to the reproductive stage of the crop. Terminal drought alone has been leading to more than 50% yield losses in chickpea. In the context of receding soil moisture, the breeding strategies should focus to enhance the maximum utilization of available soil moisture efficiently; hence breeding efforts should focus on improving root traits that enhance the efficient extraction of soil moisture. A total of 8 root traits were phenotyped for three seasons at Patancheru for gaining insights into the root system (Table S2). Association analysis identified 15 markers significantly associated with 5 root traits (Root dry weight, RDW; root length density, RLD; root surface area, RSA; root volume, RV and rooting depth, RDp) with PVE ranging from 8.25–22.41%. Among them, 7 markers showed significant association with single trait (RDp) and 2 markers (NCPGR7, DR_237) showed associations with more than one trait (Table 1). Hence, these two markers are believed to be associated with co-localized/pleiotropic QTLs. The co-localization of specific genes/QTLs could be a better way to understand the molecular basis of drought tolerance or of traits related to drought response. The presence of several co-localized/pleiotropic QTLs verified the complex quantitative nature of drought tolerance in chickpea and allowed the identification of some important genomic regions for traits related to drought tolerance. The markers associated with more than one trait may be efficiently utilized in improvement of more than one trait simultaneously through marker assisted selection (MAS). Till date there are no reports of association studies in the case of chickpea, however the association studies in other crop species especially in cereals such as maize [32], barley [33], sorghum [34] and wheat [35] have revealed that the linkage based QTL analyses can be complemented by LD based association studies.

#### Morphological traits

Among morphological traits, significant marker trait associations were identified only for plant height (PHT), shoot dry weight (SDW), apical secondary branches (ASB) and basal primary branches (BPB). A total of 47 significant MTAs were identified in the present study for the morphological traits that explained 7.77–38.01% phenotypic variation. Notably, 35 (74.49%) MTAs identified were for PHT (Table 1). Among 35 markers (2 SNPs, 2 SSRs, 29 DArT loci and 2 gene-based SNPs) that were associated with PHT, TA28 locus explained maximum phenotypic variation (38.01%).

#### Phenolological traits

Three phenological traits days to 50% flowering (DF; refers to the day when more than 50% of the plants in a plot initiated flowering), days to maturity (DM) and flowering days (FD; refers to the number of days from the start of flowering to cessation of flowering) were phenotyped for 7–11 seasons in 1–5 locations (Kanpur, Patancheru, Debre Zeit, Egerton and Nairobi). The variation in crop duration is known to affect the seed yield under both drought and heat stresses [5]. Among three traits, 5 significant MTAs were identified for DM and 2 significant MTAs for FD. CaSTMS2 marker associated with FD explained highest phenotypic variation (96.55%; Table 1). Among 4 markers (TA14, CKaM1056, cpPb-489599, ASR391(C/T) and ASR447(C/T)), associated with DM, TA14 explained maximum phenotypic variation (79.31%). Further, among phenological traits high heritability was observed in case of DF followed by DM and FD (Table S3), indicating the possibility of selecting phenotypes required for various target environments. Early maturity is one of the crop adoption strategies to escape drought and heat stresses [36]. Markers that are associated with DM and have significant negative effect on the trait will serve in selection of genotypes with early maturity, thus enhance drought tolerance. Conventionally, late sowing is recommended to overcome heat stress [36]; however, the unpredictable climatic condition may lead to yield losses significantly in case of late planting. Hence, identification of markers associated with DF and DM can be deployed for developing lines that escape drought.

#### Yield and yield related traits

A total of 13 yield and yield related traits (100-seed weight, 100SDW; Biomass, BM; Harvest index, HI; Yield, YLD; Podm^−2^, POD; Podsplant^−1^, PPP; Seedm^−2^, SPM, Seedpod^−1^, SPP; shoot day^−1^, PDS and Total dry matter weight, TDM) were studied in 1–7 seasons, 1–2 environments at two location in India (Patancheru and Kanpur) and three locations in Africa (Debre Zeit, Nairobi and Egerton). A total of 221 significant MTAs were identified in the present study for the yield and yield component traits that explained 4.23–55.42% phenotypic variation. Notably, 70 (22.43%) MTAs identified were for 100SDW (Table 1). Among 26 markers (9 SNPs, 6 SSRs, 6 DArT loci and 5 gene-based SNPs) that were associated with 100SDW, TA71 locus explained maximum phenotypic variation (88.34%). Further, among 5 gene-based SNP markers [AM_192, ASR_192_290, Ca_Cap2promo, CAP2promo98(C/G) and DR_237], CAP2promo98(C/G) explained maximum phenotypic variation (36.95%). The heritability of 100SDW was more than 0.9 across all environments and locations, except for Kanpur (0.671) under heat stress environment (Table S3). Thirty four significant MTAs were found in case of SPM (8.06–55.42%, PVE), 32 in case of YLD (11.43–29.03%, PVE), 16 in case of HI (4.23–15.53%, PVE), 11 in case of BM (16.34–18.99% PVE), 13 in case of SPP (7.72–17.75% PVE), 10 for POD (9.18–22.05% PVE), 9 for TDM (8.84–12.88% PVE), 6 for PPP (8.27–50.44% PVE), and 1 for PDS (10.09%, PVE).

#### Transpiration efficiency related traits

SPAD chlorophyll meter reading (SCMR) and δ^13^C are the transpiration efficiency related traits phenotyped in the present study. However, 22 significant MTAs were identified for δ^13^C explaining phenotypic variation ranging from 7.81–34.77%. The δ^13^C is considered as an indirect measure of transpiration efficiency and MTA identified in the present study can be used for improving transpiration efficiency. The heritability values ranges from 0.65 to 0.71 for δ^13^C, indicating that this can be an ideal surrogate to breed for transpiration efficiency in chickpea.

**Figure 3:**
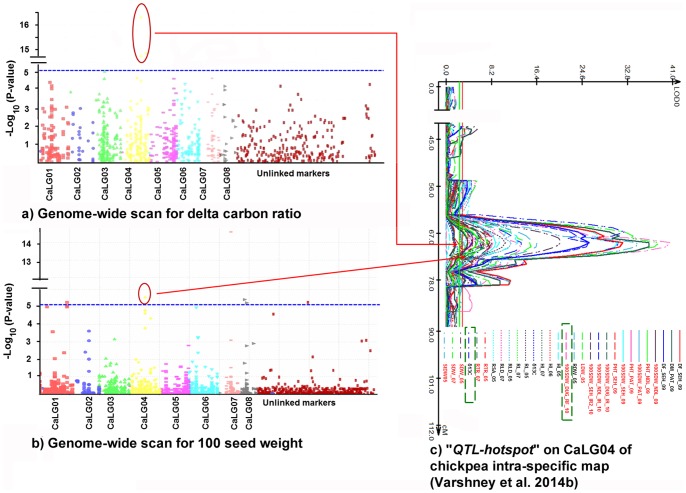
Significant marker trait associations (MTAs) for δ^13^C and 100 seed weight mapped on to “*QTL-hotspot*” on CaLG04 of intra-specific map of chickpea. (a) Genome wide association scan for δ13C; the Y-axis represent -log_10_(P) values of the P-value of the MTAs, while linkage groups are indicated on X-axis. (b) Genome wide association scan for 100SDW. (c) “*QTL-hotspot*” on CaLG04 of chickpea intra-specific genetic map harboring QTLs for drought tolerance related traits. Significant MTAs for 100SDW and δ13C falling in the QTL region are indicated using the arrows in red, the traits are indicated using dotted rectangles in green.

Interestingly, the MTAs reported in the present study for δ^13^C (Fig 3a) and 100SDW (Fig 3b) were falling in the “*QTL-hotspot*” region reported on CaLG04 of intra-specific genetic map ([37]; Fig 3c), that reemphasizes the significance of QTLs detected that can be deployed in molecular breeding for trait improvement.

### Candidate gene associations

A number of genes involved in plant drought responses and tolerance have been identified in model crops [38]. The candidate genes for drought tolerance defined by sequence variants and the phenotypic data on drought and heat tolerance related traits obtained on the reference set was employed to find the association between trait and genes. A total of 113 candidate gene-based SNPs identified in 10 candidate genes were used for association analysis as result, 18 SNPs from 5 genes (*ERECTA*, 11 SNPs; *ASR*, 4 SNPs; *AMADH*, 1 SNP; *CAP2* promoter, 1 SNP and *DREB*, 1 SNP), were significantly associated with different traits. Interestingly all 11 SNPs in ERECTA gene were significantly associated with 100SDW. Furthermore, the sole SNP found in case of CAP2 promoter was detected be associated with 100SDW. AMADH192 gene-based SNP marker is significantly associated with 100SDW, explaining 25.71% phenotypic variation. The role of the gene *AMADH*, in response to stress caused by mechanical damage was evaluated by Petřivalský et al. [39]. This gene is also expected to play a role in physiological processes related to polyamine degradation, converting 4-aminobutanal to GABA [38]. Protective role of *AMADH* in response to abiotic stress is also confirmed during the present study. The *AMADH* gene is found to be associated with four stress related root traits, deciphering the importance of this gene in abiotic stress response.

The gene-based SNP markers with significant MTAs were used to identify the coordinates on the chickpea genome and the amino acid changes due the SNPs were identified (Table 2). Among 11 SNPs in ERECTA7f fragment, 2 SNPs (ERECTA7f_33 and ERECTA7f_682) were found in the CDS regions, however, none of the SNPs had any effect on the change in amino acid. While in case of *ASR* gene all three SNPs that had significant associations were in the CDS region. Two SNPs ASR_209 and ASR_261 were associated with δ13C altered the amino acid they code for. In the case of ASR_209, asparagine is changed to glutamic acid while in the case of ASR_261 valine is changed to lysine. Interestingly, asparagine synthase has been reported to be negatively correlated with most performance parameters under drought stress in case of rice [40] and the presence of glutamic acid has been reported to increase the drought tolerance. Further, enhanced lysine content has been demonstrated to possess enhanced drought tolerance in maize (http://www.echocommunity.org/resource/resmgr/a_to_z/azch3gra.htm). In addition lysine increases the chlorophyll content in leaves and thus enhances drought tolerance. Among the candidate genes used in the present study, genes like *ERECTA* and *DREB* were reported in the “*QTL-hotspot*” region on CaLG04 of chickpea [14]. In addition *ERECTA* gene was also reported to enhance transpiration efficiency [41] and water use efficiency and transpiration efficiency in *Arabidopsis* [42] and *DREB* has been reported to play important role in abiotic stress response in *Medicago* [43]. Hence, the availability of the physical map [14], consensus genetic map [15] and the MTAs established in the present study can be effectively deployed for cloning of these important genes for enhancing the drought tolerance in chickpea.

**Table 2:** Candidate gene- based marker trait associations

Trait	Marker associated	SNP position	Major allele	Minor allele	Chromos-ome	Position on Genome	Feature	Amino acid	Altered amino acid
100SDW	ERECTA7f_33	33	A	G	Ca4	44785692	CDS	Leucine	Leucine
	ERECTA7f_187	187	C	A	Ca4	44785846	Intron	-	-
	ERECTA7f_304	304	C	T	Ca4	44785949	intron	-	-
	ERECTA7f_317	317	T	C	Ca4	44785962	intron	-	-
	ERECTA7f_424	424	G	C	Ca4	44786069	intron	-	-
	ERECTA7f_558	558	G	T	Ca4	44786203	intron	-	-
	ERECTA7f_587	587	A	C	Ca4	44786232	intron	-	-
	ERECTA7f_601	601	C	T	Ca4	44786246	intron	-	-
	ERECTA7f_682	682	T	C	Ca4	44786327	CDS	Phenyl alanine	Phenyl alanine
	ERECTA7f_741	741	A	G	Ca4	44786386	intron	-	-
	ERECTA7f_883	883	A	G	Ca4	44786528	intron	-	-
RV, RSA, PODM and 100SDW	DREB_237	237	C	G	Scaffold134	275138	Non-genic	-	-
100 SDW	AMDH_192	192	C	T	Ca7	9152243	Intron	-	-
FD	CAP2prom_166	185	C	T	Scaffold134	275092	Non-genic	-	-
δ^13^C	ASR_209	209	G	A	Ca4	11451303	CDS	Asperagine	Glutamic acid
δ^13^C	ASR_261	261	G	A	Ca4	11451251	CDS	Valine	Lysine
DM	ASR_315	315	C	G	Ca4	11451197	CDS	Serine	Aspartic acid

### Marker-trait associations for molecular breeding

The ultimate aim of breeding is to obtain higher yields under stress conditions. In the present study, a total of 159 MTAs were identified with PVE >25% for 4 important traits. A total of 38 significant allele effects for these 8 traits were identified associated with 9 markers showing significant impact on these traits while 9 markers were found to be associated with multiple traits (Table 3). All these associated markers and identified genotypes with favorable alleles can be deployed after validation for improving above mentioned traits through molecular breeding.

**Table 3:** Significant marker trait associations with >25% phenotypic variations and their effects on the trait

Trait	Season	Marker		Allele	PVE	Effect	Mean phenotypic value		
		Name	Type				Genotypes with +ve locus	Genotypes with -ve locus	Population mean
100SDW	EIAR_RF_2008	cpPb-677136	DArT	0	35.72	3.36	43.12	19.64	19.62
100SDW	EIAR_RF_2009	Ca_Cap2promo	GB-SNP*	C:C	35.41	−9.21	25.17	16.47	19.07
100SDW	EIAR_RF_2009	CKaM0804	SNP	T:T	35.41	−8.97	25.80	16.17	19.03
100SDW	EIAR_RF_2009	DREB_237	GB-SNP	C:C	35.41	−10.90	16.51	25.36	18.33
100SDW	PAT_RF_2003	CKaM0804	SNP	C:C	31.45	7.45	21.90	14.61	16.76
100SDW	PAT_IR_2001	Ca_Cap2promo	GB-SNP	G:G	31.27	6.91	20.44	13.95	15.50
100SDW	PAT_IR_2001	CKaM0804	SNP	C:C	31.27	6.92	20.84	13.29	15.47
100SDW	PAT_IR_2008	DR_237	GB-SNP	C:C	30.47	−6.19	13.15	18.35	14.14
100SDW	PAT_RF_2001	CKaM0804	SNP	C:C	29.46	7.81	23.70	15.38	17.81
100SDW	EIAR_RF_2008	DR_237	GB-SNP	C:C	28.96	−8.18	17.28	22.90	18.47
100SDW	PAT_RF_2001	TA200	SSR	286∶286	27.80	−2.80	16.38	18.21	17.91
100SDW	100SDW_RF_2008	Ca_Cap2promo	GB-SNP	C:C	27.67	−5.85	20.85	14.48	16.13
100SDW	100SDW -RF	CAP2prom98(C/G)	GB-SNP	C:C	27.30	−6.90	19.66	15.91	16.92
100SDW	100SDW_RF_2009	DR_237	GB-SNP	C:C	26.18	−5.21	15.12	22.79	16.34
PROD	PROD_MINI_ENV5	CKaM1144	SNP	A:A	50.45	1.79	5.60	3.52	5.62
SPM	SeedNom-2_IR_2008	ERECTA7f_741(A/G)	GB-SNP	A:A	55.43	975.59	2519.50	2421.90	2498.73
YLD	YKGH_MINI_ENV5	CKaM1144	SNP	A:A	28.10	148.10	643.65	430.03	641.54
YLD	Yldkghaday_RF_2009	CKaM0923	SNP	C:C	28.08	5.94	33.52	28.63	33.28
YLD	YKGH_MINI_ENV5	cpPb-491124	DArT	1	27.53	−138.96	653.49	646.54	646.46

*GB-SNP =  Gene based SNP.

## Conclusion

To understand the genetic basis of tolerance to drought and heat stresses in chickpea, a comprehensive association mapping approach has been undertaken. As a result, 312 MTAs were identified and maximum number of MTAs (70) was identified for 100-seed weight. In the present study, the pairwise LD estimated using the squared-allele frequency correlations (r^2^; when r^2^<0.20) was found to decay rapidly with the genetic distance of 5 cM (Fig 2), when r^2^<0.1 LD decay was found to decay at a genetic distance of 20 cM. Among 113 gene-based SNPs, 6 SNPs in *ASR*, 3 SNPs each in *DHN* and *DREB* were also found to have significant associations with traits like 100-seed weight, δ^13^C, plant height, root dry weight, pods per plant and yield under stressed conditions. This study provides significant MTAs for drought and heat tolerance in chickpea that can be used, after validation, in molecular breeding for developing superior varieties with enhanced drought and heat tolerance.

## Materials and Methods

### Plant material

A chickpea reference set, comprising of 300 accessions (including 267 landraces, 13 advanced lines and cultivars, 7 wild *Cicer* accessions, and 13 accessions with unknown biological status) defined based on molecular characterization of global composite collection [26], captured 1,315 (78%) of the 1,683 composite collection alleles was employed in the present study (Table S1). The mini-core collection of chickpea, is a subset of reference set, comprises of 211 accessions [27].

### DNA isolation and quantification

The DNA was isolated from the tender leaf tissues of 15 day old seedlings as per Cuc and colleagues [44]. The quality and quantity of DNA was checked on 0.8% agarose gel using λ-DNA standard. The DNA was normalized to 5 ng/ µl for further use.

### Genotyping of reference set

In addition to the genotyping data for 35 SSR markers generated previously [26], 15,360 DArT loci and 651 SNP markers using KASPar assays were genotyped on the reference set.

### DArT genotyping

The reference set was genotyped with the 15,360-clone DArT arrays developed by Thudi et al. [10]. In brief, genomic representations for genotyping were prepared by the complexity reduction method described by Yang and colleagues [45]. Briefly, ca. 100 ng of DNA of reference set were digested with restriction enzymes *Pst*I and *Hae*III (New England Biolabs, USA) and the *Pst*I adapter was simultaneously ligated. One μl of restriction/ligation reaction served as a template in a 50 µl amplification reaction with *Pst*I+0 primer. Adaptor and primer sequences and cycling conditions are given in the earlier study [46]. Arrays were hybridized with fluorescently labeled targets from all genotypes used for the array development [45], [46]. After overnight hybridization at 62°C, the slides were washed and scanned with a Tecan LS300 confocal laser scanner (Grödig, Salzburg, Austria). Individual samples were processed identically to samples for marker discovery and with similar marker quality thresholds in DArTsoft analysis [10].

### SNP genotyping

Based on high PIC values and distribution of SNPs on the chickpea genome, 651 KASPar assays for the targeted SNPs were selected from [11] for genotyping at LGC Genomics, UK. Details on principle and procedure of the assays are available at http://www.kbioscience.co.uk/reagents/KASP_manual.pdf and http://www.kbioscience.co.uk/download/KASP.swf. SNPViewer was employed for SNP calling.

### Candidate gene selection, sequencing and SNP identification

The candidate drought responsive genes were genotyped either on reference set or on the mini-core collection (Table S7). The candidate genes like abscisic acid stress and ripening gene (*ASR*), drought responsive element binding protein (*DREB*) gene, *ERECTA*, sucrose synthase (*SuSy*), sucrose phosphate synthase (*SPS*), dehydrin (*DHN*), aminoaldehyde dehydrogenase (*AMADH*), *AKIN* (SNF1 related protein kinase), MYB transcription factor, responsible for drought tolerance were identified based on prior information of involvement of the genes in drought tolerance mechanism in other crop species. In order to identify the above mentioned candidate genes in chickpea, the sequences were downloaded either from chickpea or from related legume species like *Medicago*, which is phylogenetically related to chickpea.

The candidate genes were amplified on the reference/mini-core collection. The PCR amplicons were purified using Exonuclease I and 1 U of shrimp alkaline phosphatase (SAP) per 5 µl of PCR product. The Exo/SAP added PCR products were products were incubated for 45 min at 37°C followed by denaturing at 80°C for 15 min in the thermal cycler for deactivating unused Exonuclease enzyme. The Exo/SAP treated amplicons were mixed with 1 µl of BigDye Terminator V3.1 (Applied Biosystems, California, USA), 2 µl of 5× sequencing dilution buffer and 3.2 µM of primer (forward and reverse separately) and the volume was made to 10 µl. The sequencing PCR profile included an initial denaturation of 96°C for 10 sec, 50°C for 5 sec, and 60°C for 4 min. Before sequencing, the PCR products were treated with 2.5 µl of 125 mM EDTA and 25 µl of absolute ethanol and incubated for 15 min at room temperature to precipitate the DNA. The plate containing the PCR product was centrifuged at 4000 rpm for 30 min at 4°C. The Ethanol/EDTA mix was poured off by inverting the plate, without losing the pellet. To each well, 60 µl of 70% ethanol was added and again spun at 4000 rpm for 20 min at 4°C. The ethanol was poured off as earlier. The plate was air-dried and 10 µl of HiDi formamide (Applied Biosystems, California, USA) was added and the products were denatured (94°C for 5 min, then immediately cooled to 4°C for 5 min) and sequenced using an ABI3700 automated sequencer (Applied Biosystems, California, USA). The large-scale sequencing of candidate genes across 300 genotypes of reference set was carried out at MACROGEN, Korea using BigDye terminator cycle sequencing chemistry.

The gene sequences were subjected to BLAST against chickpea reference genome assembly [1] and the genome coordinates of these genes were determined. Using the genome coordinates for each genes, the features and the amino acid changes due to the SNPs were determined using SNPeff tool (http://snpeff.sourceforge.net/).

#### Phenotyping of reference set/mini-core collection

In the present study, the reference set/mini-core collection was phenotyped for 34 traits in 5 locations (Patancheru, Kanpur, Nairobi, Debre Zeit and Egerton) in three countries (India, Ethiopia and Kenya), in 1–5 environments (cylinder culture, CC; rainfed, RF; irrigated, IR; normal and heat stress environments) in 2–3 replications. The detailed information on the traits phenotyped, locations and seasons are provided in Table S2.

### Root traits

The reference set was phenotyped for drought tolerance related root traits (root length, RL, cm plant^−1^; root length density, RLD, cm-cm^−3^; root dry weight, RDW, g plant^−1^; rooting depth, RDp, cm; root surface area, RSA, cm^2^ plant^−1^; root volume, RV, cm^3^; ratio between RDW, and total dry weight, RTR, %; Average diameter, AVD, mm; projected area, PRA, cm^2^ plant^−1^) for three seasons (2007–08, 2008–09, 2010–11) in cylinder culture in three replications using semi-automated high-throughput precise phenotyping facility at ICRISAT, Patancheru as described earlier [47].

### Morphological traits

The reference set/mini core collection was phenotyped for morphological traits like shoot dry weight (SDW, g plant^−1^), for three seasons (2007–08, 2008–09, 2010–11) in cylinder culture in three replications at Patancheru. In addition, morphological traits like plant height (PHT, cm), plant width (PWD, cm), apical primary branches (APB), apical secondary branches (ASB), basal primary branches (BPB), basal secondary branches (SBS), Tertiary branches (TB) in 1–5 locations and 1–13 seasons (Table S2) in rainfed and or irrigated environments in 2–3 replications.

### Phenological traits

Phenological traits like days to 50% flowering (DF), days to maturity (DM), and flowering days (FD) were phenotyped on reference/mini-core collection in 1–5 locations and 1–7 seasons (Table S2).

### Yield and yield component traits

The reference set/mini-core collection was phenotyped for yield and yield related traits like seeds pod^−1^ (SPD), pods plant^−1^ (PPP), 100-seed weight (100SDW, g or kg/ha), yield (YLD, kg/ha), yield plant^−1^ (YPP, g), biomass (BM, kg), harvest index (HI, %), seeds m^−2^ (SPM), total dry matter weight (TDM, g).

In addition transpiration efficiency related traits like delta carbon ratio (δ^13^C), and SPAD chlorophyll meter reading (during 2004–05 and 2005–06) specific leaf area were also phenotyped (Table S2).

### Heat tolerance phenotyping

A set of 280 accessions from reference set were phenotyped during the post-rainy 2009–10 in two sowing dates (normal and late sowing) on a Vertisol at Patancheru and in an Inceptisol (sandy loam) at Kanpur. The alpha lattice design was adopted and evaluated in three replications at both the locations.

## Data Analysis

### Statistical analysis

For each trait, data was analyzed using analysis of variance (ANOVA) by using SAS General Linear Model (GLM) procedure [48] considering all effects as fixed. Along with ANOVA results, least square means (LSM; genotype as fixed effect), standard error of differences (SED), least significant difference (LSD) and descriptive statics like coefficient of variation (CV) and grand mean (GM) were calculated. Considering genotype as random, best linear unbiased predictors (BLUPs) were estimated by using SAS MIXED procedure [48]. Correlation coefficients among different traits were calculated by Karl Pearson's method using SAS CORR procedure [48]. Considered genotype as random in the statistical model and variance components were estimated for each effect, which are used for calculating heritability.

### Marker attributes and diversity analysis

The marker attributes like major allele frequency, gene diversity and PIC value for all markers was computed using PowerMarker ver. 3.25 [49]. Neighbor joining trees were constructed using SSR, DArT and SNP markers data independently and combining all the data using DARwin ver. 5.0.158 [50] (http://darwin.cirad.fr/darwin). Neighbor joining trees were viewed employing Dendroscope ver. 3.2.2 [51] (http://ab.inf.uni-tuebingen.de/data/software/dendroscope3).

### Population structure

A set of 85 DArT loci uniformly distributed across the chickpea genome [10] were used to understand the genetic structure and number of sub-populations in the reference set employing STRUCTURE version 2.3.1 [29] (http://pritch.bsd.uchicago.edu/structure.html) was employed. For this, the number of sub-populations (K) was presumed as 1 to 15, and each was repeated two times. For each run, burn-in and iterations were set to 1,00,000 and 2,00,000 respectively, and admixture and correlated allele frequencies was used. The run with maximum likelihood was used to assign individual genotypes into sub-population.

### Association analysis

The SNP, DArT and SSR markers data set from the reference set was used to generate a matrix of similarity between each pair of genotypes in the study (the K matrix) using the program TASSEL. The Q and K matrices were used to correct for the effects of population substructure in the association panel which can cause false positive associations. Using the Q and K matrices as a covariate, markers were tested for association with each phenotype using the program TASSEL (http://www.maizegenetics.net). A Mixed Linear Model (MLM) analysis with optimum compression with P3D method [52] was used in regression, to allow for multiple testing effects. A mixed model approach implemented in EMMA 22 was used to correct the confounding of population structure.

## Supporting Information

Figure S1 **Population structure and genetic relationships among the chickpea reference set.** a) Structure of sub-populations at different K values ranging from 2–15 b) Comparison of genetic relationships revealed by SSR, DArT and SNP markers clearly indicated three major clusters.
(TIF)

Table S1 **Information on the association panel used in the present study.**
(XLSX)

Table S2 **Summary of phenotyping data generated on reference set/mini-core collection.**
(XLSX)

Table S3 **Summary statistics of root traits, morphological, phenological, transpiration efficiency related traits, yield and yield component traits evaluated on reference set and/mini-core collection.**
(XLSX)

Table S4 **Major allele frequency, gene diversity and PIC content of 35 SSR loci used in the study.**
(XLSX)

Table S5 **Major allele frequency, gene diversity and PIC content of 1,156 DArT loci used in the study.**
(XLSX)

Table S6 **Number of alleles and PIC values of 381 SNP markers used in the study.**
(XLSX)

Table S7 **Candidate genes used for association mapping.**
(XLSX)
