# Correction: Genetic Dissection of Drought and Heat Tolerance in Chickpea through Genome-Wide and Candidate Gene-Based Association Mapping Approaches

**DOI:** 10.1371/journal.pone.0175609

**Published:** 2017-04-06

**Authors:** Mahendar Thudi, Hari D. Upadhyaya, Abhishek Rathore, Pooran Mal Gaur, Lakshmanan Krishnamurthy, Manish Roorkiwal, Spurthi N. Nayak, Sushil Kumar Chaturvedi, Partha Sarathi Basu, N. V. P. R. Gangarao, Asnake Fikre, Paul Kimurto, Prakash C. Sharma, M. S. Sheshashayee, Satoshi Tobita, Junichi Kashiwagi, Osamu Ito, Andrzej Killian, Rajeev Kumar Varshne

There are multiple errors throughout Table S3 in the minimum, mean, and maximum values for the Root length density, Plant Height, Days to 50% flowering, 100 seed weight, Biomass, pods per plant, seeds per pod, seeds per plant, and dry matter weight traits. Please see the corrected Table S3 attached to this document as a Supporting Information file. For more information concerning the error, please contact the corresponding author, Rajeev Kumar Varshney (R.K.Varshney@CGIAR.ORG) or the first author, Mahendar Thudi (T.Mahendar@CGIAR.ORG).

## Supporting information

S3 File Summary statistics of root traits, morphological, phenological, transpiration efficiency related traits, yield and yield component traits evaluated on reference set and/mini-core collection. (XLSX)
