# Supplementary figures and images for: Genetic Dissection of Drought and Heat Tolerance in Chickpea through Genome-Wide and Candidate Gene-Based Association Mapping Approaches
Source: PLoS One. 2014 May 6;9(5):e96758. doi: 10.1371/journal.pone.0096758 (PMC4011848; doi:10.1371/journal.pone.0096758)

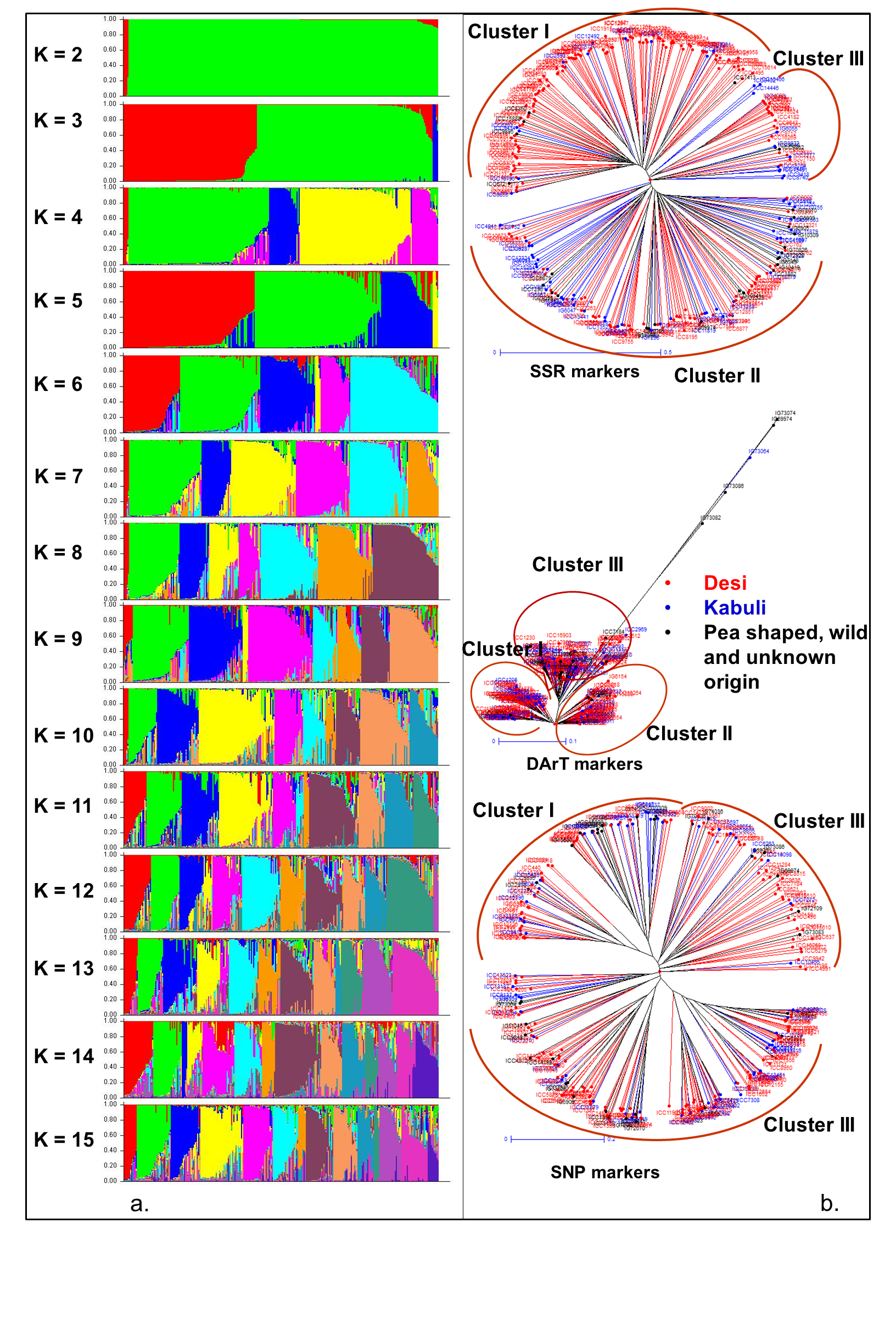

Supplement: Figure S1 — Population structure and genetic relationships among the chickpea reference set. a) Structure of sub-populations at different K values ranging from 2–15 b) Comparison of genetic relationships revealed by SSR, DArT and SNP markers clearly indicated three major clusters. (TIF) [file pone.0096758.s001.tif]
